# Tetracycline Gene Transfer in *Staphylococcus xylosus in situ* During Sausage Fermentation

**DOI:** 10.3389/fmicb.2019.00392

**Published:** 2019-03-06

**Authors:** Sabine Leroy, Souad Christieans, Régine Talon

**Affiliations:** ^1^ Université Clermont Auvergne, INRA, MEDiS, Clermont-Ferrand, France; ^2^ ADIV, ZAC des Gravanches, Clermont-Ferrand, France

**Keywords:** *S. xylosus*, antibiotic resistance, tetracycline, transfer, sausage

## Abstract

The presence of determinants of resistance to antibiotics can constitute a possible safety hazard in coagulase-negative staphylococci (CNS), which are widely present in food of animal origin. Among CNS, *S. xylosus* is a species frequently isolated from fermented meat products. Resistance to tetracycline was found to be one of the most distributed resistances occurring in *S. xylosus* strains isolated from fermented sausages. We evaluated the transfer of tetracycline resistance *in vitro* and *in situ* between *S. xylosus* strains. We selected three strains isolated from dry fermented sausages, resistant to tetracycline but not to minocycline, their resistance occurring by a mechanism of active efflux encoded by the *tetK* gene. Only one strain was able to transfer its tetracycline resistance to a recipient strain initially susceptible and plasmid-free using a filter mating procedure. Transfer of tetracycline resistance was observed at very low frequencies of 3.4 × 10^−9^ per recipient. To further investigate the transferability of this tetracycline resistance, the donor and recipient strains were tested in pilot-scale fermented sausage production. This transfer was possible but at a low rate, 1.4 × 10^−7^, and only under conditions of a high inoculation level of 10^8^ CFU/g of meat. The *tetK* gene is located on a small mobilizable plasmid close to *Staphylococcus aureus* pT181 plasmid. In conclusion, the transfer of tetracycline resistance between strains of *S. xylosus* is possible, but at a really low frequency *in vitro* and *in situ* in fermented sausages. Even if this represents a very moderate risk, it should be taken into account as required by the European approach of Qualified Presumption of Safety (QPS) and AFSSA safety recommendations, advising that strains used as starter cultures should not carry any transferable antibiotic resistance.

## Introduction

Coagulase-negative staphylococci (CNS) are widely present in microbial ecosystems of fermented foods, in particular of animal origin (Coton et al., 2010; Leroy et al., 2010; Greppi et al., 2015; Sánchez Mainar et al., 2017). Among CNS, *S. xylosus* is a ubiquitous gram-positive bacterium naturally present in these foods and commonly used as starter culture for dry fermented sausages (Coton et al., 2010; Leroy et al., 2010; Ratsimba et al., 2017). It is shown to occur in numbers of 10^6^–10^7^ CFU/g in naturally fermented meat products and in inoculated ones.

Tetracycline is a broad-spectrum antibiotic that has been widely used since the 1940s (Chopra and Roberts, 2001). As a consequence of its use in animal husbandry, antibiotic-resistant pathogenic and commensal bacteria have been detected in various animal products including fermented foods derived from meat and milk (Devirgiliis et al., 2011; Li et al., 2016; Chon et al., 2016; Anisimova and Yarullina, 2018; Ed-Dra et al., 2018; Lüdin et al., 2018; Wu et al., 2018). Among these bacteria, CNS can be a reservoir of antibiotic-resistant bacteria (Malik et al., 2005; Even et al., 2010; Talon and Leroy, 2011). In several studies, resistance to tetracycline was found to be the most distributed resistance occurring in *S. xylosus* strains isolated from the food chain. Thus, the 12 strains of *S. xylosus* collected from the production chain of swine meat commodities (feces, feed, meat, sausage) were resistant to tetracycline (Simeoni et al., 2008). This high prevalence of resistance was also true for *S. xylosus* isolated from domestic animals (oropharyngeal, rectal samples) (Bhargava and Zhang, 2012). Resistance to tetracycline ranging from 12 to 38% was recorded for *S. xylosus* associated with fermented sausages, cheeses, or meat starter cultures (Mauriello et al., 2000; Kastner et al., 2006; Martín et al., 2006; Resch et al., 2008; Even et al., 2010; Marty et al., 2012; Chajęcka-Wierzchowska et al., 2015).

The tetracycline resistome represented by more than 40 determinants falls in three categories, ribosomal protection proteins, active efflux pumps, and enzymatic inactivation (Thaker et al., 2010; van Hoek et al., 2011). In staphylococci resistance can occur by ribosome protection encoded by *tetM*, *tetO*, *tetS*, and *tetW* genes, by efflux pumps encoded by *tetK*, *tetL, tet38*, and *tet42* genes, and by an unknown mechanism encoded by *tetU* (Roberts, 1996; Schwarz et al., 1998; Malik et al., 2005; van Hoek et al., 2011). The gene *tetM* was mostly identified in *S. xylosus* isolated from the skin of pigs (Schwarz and Noble, 1994), the production chain of swine meat commodities (Simeoni et al., 2008) and domestic animals (Bhargava and Zhang, 2012), but it was also found in ready-to-eat food of animal origin (Chajęcka-Wierzchowska et al., 2015). The conjugative transposon Tn*916* is often associated with *tetM* (Chopra and Roberts, 2001; Chajęcka-Wierzchowska et al., 2015). However, *tetK* was found to be the dominant mechanism in *S. xylosus* (Kastner et al., 2006; Resch et al., 2008; Even et al., 2010; Marty et al., 2012). Gram-positive efflux genes are associated with small potentially transmissible plasmids (Schwarz and Noble, 1994; Chopra and Roberts, 2001).

The consumption of fermented foods such as fermented sausages with high levels of *S. xylosus* resulted in ingestion of high amounts of living bacteria. Antibiotic resistance determinants present in *S. xylosus* strains naturally and frequently present in fermented meat products lead to the question: Could these strains act as reservoirs for antibiotic resistance gene spreading in starter culture strains? Thus, the objective of this work was to study *in vitro* and *in situ* in fermented sausages the risk and frequency of intra-specific horizontal transfer of tetracycline resistance gene.

## Materials and Methods

### Bacterial Strains and Growth Conditions

Four strains of *S. xylosus* were used. Three strains isolated from dry fermented sausages are resistant to tetracycline and sensitive to minocycline, their resistance occurring by a mechanism of active efflux encoded by the *tetK* gene as determined by hybridization of a diagnostic microarray including 10 *tet* gene probes commonly found in Gram*-*positive bacteria, i.e., *tetA*, *tetB*, *tetK*, *tetL*, *tetM*, *tetP*, *tetS*, *tetT*, *tetU*, and *tetW* (Even et al., 2010). The C2a strain is derived from the type strain DSM20267 cured of its endogenous plasmid (Götz et al., 1983). This strain is sensitive to tetracycline and resistant to rifampicin. We selected a spontaneous fusidic acid-resistant mutant from the C2a strain as a recipient strain.

The *S. xylosus* strains were cultured in Brain Heart Infusion (BHI, Difco) under aerobic conditions (1:10 volume to flask ratio, 150 rpm) or on BHI Agar (Difco) at 37°C. The BHI medium, when required, was supplemented with antibiotics at the following concentrations: 10 μg/ml tetracycline (Tet), 25 μg/ml rifampicin (Rif), and/or 25 μg/ml fusidic acid (Fus) (Sigma-Aldrich).

### Transfer *in vitro*

Transfer experiments were performed by filter mating. The donor and recipient strains were cultivated separately overnight with the appropriate resistance selection to reach about 8 × 10^8^ CFU/ml. Cells were mixed in a donor/recipient ratio of 1:3 or 1:10. Typically, 350 or 100 μl of donor and 700 or 900 μl of recipient, respectively, were pelleted and suspended in 400 μl of BHI and dropped onto 0.45 μm cellulose nitrate membrane filters (Whatman). The filters were incubated on BHI agar plates for 18 h at 37°C. After mating, cells were recovered from the filters by vortexing in 1 ml of BHI. Transconjugants were selected by plating serial dilutions of the mating suspension on BHI agar supplemented with Tet, Rif, and Fus. The plates were incubated for 48 h at 37°C. After incubation, the resulting colonies were picked up and replicated on BHI containing Tet, Rif, and Fus. Likewise, donor and recipient strains were plated as control onto BHI agar containing Tet or Rif and Fus, respectively. The frequency of transfer was expressed as the number of conjugants per recipient. Presumptive transconjugants were confirmed by antibiotic disk diffusion, PCR-based detection of *tetK* gene, plasmid profiling, and PFGE typing as described below.

### Transfer *in situ* in Fermented Sausages

Batter was prepared by grinding raw materials (lean pork 80%, pork back fat 20%) to obtain particles of 6 mm diameter and was seasoned with dextrose (0.5%), salt (2.6%), saltpeter (0.03%), and pepper (0.15%). It was inoculated with starter culture *Lactobacillus sakei* 10^6^ CFU/g. The batter was then divided into two batches that were inoculated by *S. xylosus* strains XIV 10B1 and C2a at about 10^6^ CFU/g each in batch 1 and 10^8^ CFU/g each in batch 2. The mixtures were stuffed into natural casings. After stuffing, sausages were dipped into a *Penicillium* surface suspension and hung vertically in a temperature and humidity controlled incubator (ARCOS, France) to carry on the ripening process (fermentation and drying). The products were fermented at 22/24°C for 6 days 96–94% RH (relative humidity), then dried for 24 days at 13°C/14°C and 80–82% RH.

Samples were taken at T0 (after inoculation and stuffing), T6 (end of fermentation stage), and T30 (end of drying). The pH of the samples was recorded using a pH meter MP230 (Mettler Toledo, Viroflay, France) with a pH probe (Inlab 427 penetration probe; Mettler Toledo). The water activity (aw) was measured with an aw-sprint TH500 (Novasina, Roucaire, France). Lactic acid bacteria (LAB) were enumerated on MRS agar (Oxoid) pH 5.7 incubated in anaerobic conditions at 30°C for 72 h. Staphylococci were enumerated on Chapman (Oxoid) after incubation for 24–48 h at 37°C. After counting, a replica-plating procedure was performed on BHI agar supplemented with appropriate antibiotics. Bacterial enumeration was also directly performed on BHI agar supplemented with (1) Rif and Fus, (2) Tet, or (3) Tet, Rif, and Fus. The plates were incubated for 24–48 h at 37°C. Rapid identification of *S. xylosus* was performed by direct colony PCR using species-specific primers as described (Morot-Bizot et al., 2003; Corbière Morot-Bizot et al., 2004). Presumptive transconjugants were confirmed as described below.

### Disk Diffusion Method

Antibiotic susceptibilities were evaluated by the disk diffusion method according to the guidelines of the CA-SFM (2017) with Mueller Hinton agar (Sigma-Aldrich). The following antimicrobial susceptibility test disks (Bio-Rad) were used: fusidic acid (10 μg), minocycline (30 μg), penicillin G (6 μg), rifampicin (5 μg), and tetracycline (30 μg). Inhibition zones were measured, and the susceptibility was determined as suggested by the CA-SFM standards.

### PCR Detection of *tetK Gene*

Total DNA from *S. xylosus* was isolated by the method of Marmur (1961). Approximately 10 ng of total DNA was used as a PCR template. The primers used for *tetK* amplification were GTAGCGACAATAGGTAATAGT and GTAGTGACAATAAACCTCCTA (Strommenger et al., 2003). PCR was performed in a 50 μL reaction mixture containing 5 pmol of each primer, 200 μM of each dNTP, 1.5 mM MgCl_2_, 1× Taq DNA polymerase buffer, and 0.5 U of Taq DNA polymerase (Promega). The amplification cycle was as follows: 3 min at 94°C; 30 cycles of 30 s at 94°C, 30 s at 55°C, and 30 s at 72°C; and a final extension of 4 min at 72°C. The amplification products were analyzed on 2% agarose gel and visualized by ethidium bromide. PCR product of 360-bp was further checked by sequencing.

### Plasmid Profiling and Sequencing

Plasmid DNA from *S. xylosus* was extracted using the QIAprep Miniprep Kit (Qiagen) after cell lysis with 16 μg/ml lysostaphin. The plasmid content was verified on a 0.8% agarose gel and visualized by ethidium bromide or transferred onto positively charged nylon membranes (Hybond-N+, Amersham Biosciences). Southern blot hybridization was performed using the *tetK* PCR product (360 bp long) from *S. xylosus* XIV 10B1 as a probe. The PCR product was first purified with a Qiaquick PCR purification Kit (Qiagen) and labeled using the DIG-High Prime DNA Labeling Kit (Sigma-Aldrich). Pre-hybridization and hybridization were performed with DIG Easy Hyb solution (Sigma-Aldrich) at 42°C. Washes were performed for 10 min at room temperature in 2X SSC, 0.1% sodium dodecyl sulfate (SDS), and for 30 min at 68°C in 0.1X SSC, 0.1% SDS. NBT-BCIP (Sigma-Aldrich) was used for the detection.

The plasmid sequences adjacent to the 360-bp *tetK* fragment have been recovered by inverse PCR using inversely oriented primers, IP-AM1 AAAAGATAATCCGCCCATAACA and IP-AV1 TGCTTCTGGAATGAGTTTGCT. Approximately 1 ng of plasmid content was used as a template. Inverse PCR was performed in a 50 μl reaction mixture containing 5 pmol of each primer, 200 μM of each dNTP, 1× AccuTaq buffer, and 2.5 U of AccuTaq LA DNA polymerase (Sigma-Aldrich). The amplification cycle was as follows: 5 min at 94°C; 30 cycles of 30 s at 94°C, 30 s at 55°C, and 5 min at 68°C; and a final extension of 10 min at 68°C for amplification of an expected product of about 5 kb.

The amplification product was analyzed on 0.8% agarose gel and visualized by ethidium bromide. Finally, the inverse PCR product was gel-purified with extraction from agarose using the QIAquick Gel Extraction Kit (Qiagen) for primer-walking-strategy DNA sequencing. Sequences were assembled into a contig using CAP3 (Huang and Madan, 1999) and then analyzed using the NCBI BLAST suite of programs.

### PFGE Typing

Genomic DNA of *S. xylosus* was prepared in agarose plugs as described previously (Morot-Bizot et al., 2003). The enzyme *Sma*I was used according to the manufacturer’s instructions (Promega). Digested DNA was subjected to pulsed-field gel electrophoresis (PFGE) in 1% agarose gels in 0.5× TBE buffer on a CHEF-DR III apparatus (Bio-Rad). Electrophoretic conditions were 50–100 s for 6 h and 10–30 s for 18 h at 14 C at 6 V/cm and an angle of 120°. Lambda DNA concatemers were used as molecular size markers (Promega). Gel was stained in ethidium bromide.

### Nucleotide Sequence Accession Number

The GenBank accession number of the full-length sequence of plasmid pSX10B1 with annotations is MK433518.

## Results

### Transfer *in vitro* and *in situ* in Fermented Sausages

The three *S. xylosus* strains resistant to tetracycline were used as donors, and the C2a strain was used as recipient in filter mating experiments. The transfer of tetracycline resistance was only observed in the case of a donor/recipient ratio of 1:10 and the XIV 10B1 strain as a donor. The transfer occurred at a low frequency of 3.4 × 10^−9^ per recipient, and two presumptive transconjugants (TC1 and TC2) were isolated.

To study the potential transfer of tetracycline resistance of the XIV 10B1 strain *in situ* in meat matrix, fermented sausages were made on a pilot scale mimicking the industrial process. Two batches were prepared with the same raw materials and inoculated with *L. sakei* as starter (sensitive to tetracycline), but with two different levels of *S. xylosus* (donor and recipient, ratio 1:1): one at approximately 6 log CFU/g (batch E1) and the other at 8 log CFU/g (batch E2) (Table 1). The ratio 1:1 has been chosen because plasmid transfers were observed for *Lactobacillus curvatus* during sausage fermentation (Vogel et al., 1992). The fermented sausages were analyzed over a ripening period of 30 days.

**Table 1:** Microbial and physico-chemical-characteristics of the fermented sausages over 30 days of ripening.

Selective media	Days	T0	T6	T30
Batch E1	pH	5.80 ± 0.08	5.20 ± 0.06	5.55 ± 0.12
	Aw	0.97 ± 0.01	0.95 ± 0.02	0.91 ± 0.01
MRS	Lactic acid bacteria	6.45 ± 0.26	7.68 ± 0.31	8.72 ± 0.14
Chapman	Staphylococci	6.68 ± 0.15	7.71 ± 0.15	7.46 ± 0.26
BHI + Tet	*S. xylosus* donor	5.87 ± 0.21	7.11 ± 0.14	6.87 ± 0.15
BHI + Rif + Fus	*S. xylosus* recipient	6.08 ± 0.16	5.94 ± 0.12	5.29 ± 0.16
BHI + Tet + Rif + Fus	Transconjugant	ND	ND	ND
Batch E2	pH	5.82 ± 0.05	5.25 ± 0.08	5.56 ± 0.16
	Aw	0.97 ± 0.03	0.95 ± 0.02	0.92 ± 0.02
MRS	Lactic acid bacteria	6.62 ± 0.10	7.69 ± 0.23	8.52 ± 0.15
Chapman	Staphylococci	8.32 ± 0.15	8.72 ± 0.16	8.03 ± 0.13
BHI + Tet	*S. xylosus* donor	7.92 ± 0.14	8.24 ± 0.15	7.60 ± 0.12
BHI + Rif + Fus	*S. xylosus* recipient	8.56 ± 0.16	8.33 ± 0.15	7.29 ± 0.12
BHI + Tet + Rif + Fus	Transconjugant	ND	3 colonies	1 colony

Enumeration expressed in log CFU/g.

Tet, tetracycline; Rif, rifampicin; Fus, fusidic acid; ND, not detected.

A growth of approximately two logs was recorded for lactic acid bacteria in both batches (Table 1). They grew during the fermentation and ripening steps. A drop in pH was noted during the fermentation phase followed by an increase during the ripening step in the two batches. Finally, water activity decreased during ripening in both batches (Table 1).

Enumeration of staphylococci on Chapman medium is shown in Table 1. The staphylococcal count remained close to their level of inoculation throughout the process in both batches. All randomly selected colonies were identified as *S. xylosus*. No transconjugants were isolated by replica plating of colonies grown on Chapman agar.

In batch E1, the donor strain grew during the fermentation step and then remained at the same level, while the recipient strain decreased slightly during the process (Table 1). In the condition of batch 1, no presumptive transconjugant was isolated on medium supplemented with Tet, Rif, and Fus (Table 1).

In batch E2, the donor and recipient strains remained at their level of inoculation up to the fermentation, and a slight decrease was noted at 30 days of ripening. Presumptive transconjugants were isolated on medium supplemented with Tet, Rif, and Fus after 6 days of fermentation (E2–1, E2–2, and E2–3) and after 30 days of ripening (E2–4). These presumptive transconjugants were only detected on plates inoculated from the 10^−1^ sample dilution. The frequency of the transfer can be estimated at a rate of 1.4 × 10^−7^ per recipient after 6 days of fermentation.

### Confirmation of Transconjugants

The six colonies of presumptive transconjugants were picked from selective plates and were characterized to determine if these isolates were “true” transconjugants and not mutants of donor or recipient.

Antibiotic susceptibility for presumptive transconjugants and donor and recipient strains was determined by a disk diffusion test (Table 2). All transconjugants were resistant to tetracycline and sensitive to minocycline, like the donor strain. Their resistance to rifampicin and fusidic acid was identical to those of the chromosomally resistant recipient strain. The resistance to penicillin G of the donor strain had not been transferred. Note that the transferred tetracycline resistance is stably maintained in all transconjugants in the absence of selective pressure.

**Table 2:** Antibiotic susceptibility profiles of *S. xylosus* strains determined by disk diffusion.

	Recipient	Donor	Transconjugants					
			*in vitro*		In sausages			
Disk	C2a	XIV 10B1	TC1	TC2	E2–1	E2–2	E2–3	E2–4
Tetracycline	38* (S)	10 (R)	10	11	11	11	10	10
Minocycline	38 (S)	30 (S)	36	34	38	34	36	34
Rifampicin	6 (R)	32 (S)	6	6	6	7	8	6
Fusidic acid	9 (R)	24 (S)	9	10	10	8	8	8
Penicillin G	34 (S)	14 (R)	34	33	36	32	30	32

* Diameter expressed in mm.

S, sensitive; R, resistant.

**Figure 1:**
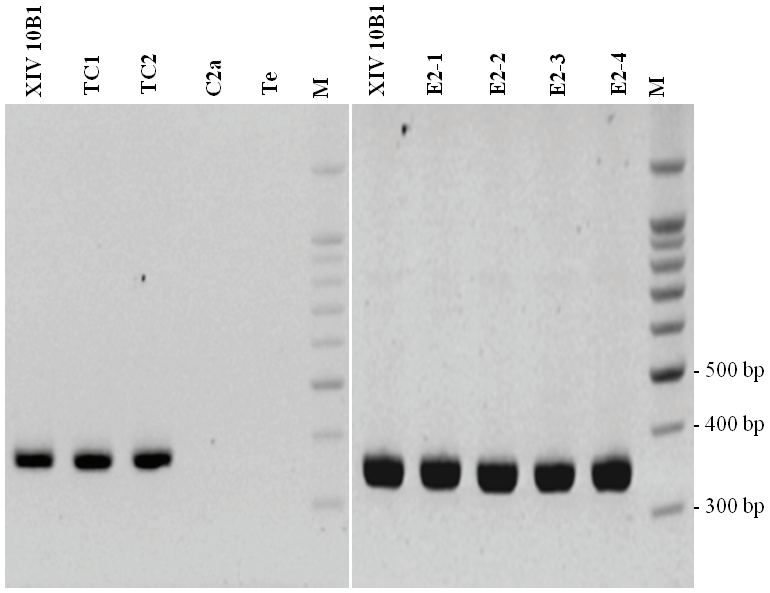
Analysis of *tetK* PCR (360 bp product). XIV 10B1: donor strain; C2a: recipient strain; TC1 and TC2: transconjugants obtained after *in vitro* mating; E2–1, E2–2, E2–3: transconjugants isolated from sausages after 6 days of fermentation; E2–4: transconjugant isolated from sausages after 30 days of ripening; Te: negative control; M: DNA 100 bp DNA ladder (Thermo Scientific).

We have shown that PCR amplification was successful in detecting *tetK* in all presumptive transconjugants as in the XIV 10B1 donor strain (Figure 1). The sequencing of the XIV 10B1 360 bp-PCR product confirmed that the PCR was specific.

**Figure 2:**
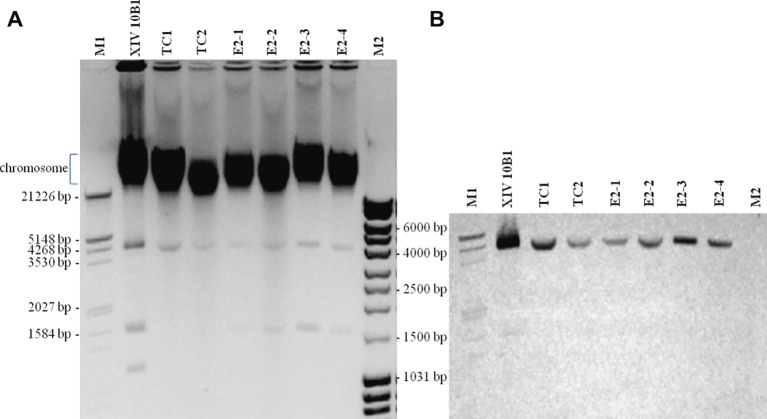
Plasmid analysis from *S. xylosus* XIV 10B1 and transconjugants. **(A)** Agarose gel stained with ethidium bromide. **(B)** Colorimetric detection of Southern blot hybridization with a DIG-labeled probe of the *tetK* gene. XIV 10B1: donor strain; TC1 and TC2: transconjugants obtained after *in vitro* mating; E2–1, E2–2, E2–3: transconjugants isolated from sausages after 6 days of fermentation; E2–4: transconjugant isolated from sausages after 30 days of ripening; M1: DNA Molecular Weight Marker III, DIG-labeled (Sigma-Aldrich); M2: MassRuler DNA ladder (Thermo Scientific).

When comparing plasmid carriage of presumptive transconjugants with the donor strain, we have shown that all transconjugants harbored a small plasmid (<5 kb) of similar size to that of the donor strain (Figure 2A). This small transmissible plasmid hybridized with the *tetK* probe (Figure 2B). We have observed in the four transconjugants obtained *in situ* the additional transfer of a smaller plasmid (Figure 2B). The complete nucleotide sequence of the plasmid carrying *tetK* was obtained. Plasmid *pSX10B1* is 4,498 bp long with 30.1% G + C content. The sequence encodes three open reading frames, which correspond to a replication initiation protein, a tetracycline resistance protein (TetK), and a plasmid recombination enzyme. It exhibits a high identity (96.5%) with the *S. aureus* COL plasmid pT181. Moreover, the amino acid sequence of TetK shows 100% identity with that of pT181.

**Figure 3:**
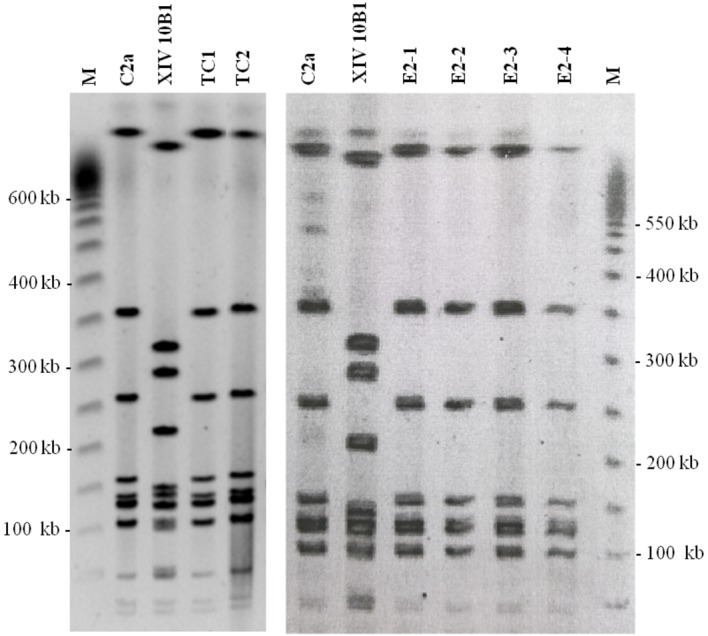
PFGE profiles from *S. xylosus* strains. C2a: recipient strain; XIV 10B1: donor strain; TC1 and TC2: transconjugants obtained after *in vitro* mating; E2–1, E2–2, E2–3: transconjugants isolated from sausages after 6 days of fermentation; E2–4: transconjugant isolated from sausages after 30 days of ripening, M: Lambda ladder (Promega).

Transconjugants, donor, and recipient were subject to PFGE analysis. The donor and recipient produced distinct patterns (Figure 3). The transconjugants displayed the same pattern as the recipient strain.

## Discussion

For several decades, the food chain can be considered the main route of transmission of antibiotic-resistant bacteria between animals and humans (Witte, 1998; Verraes et al., 2013). In the specific case of fermented products, they may be vehicles for large amounts of living bacteria (Mathur and Singh, 2005). In fermented meat products, the most important microorganisms responsible for fermentation are lactic acid bacteria (LAB) and coagulase negative staphylococci (CNS). The antibiotic resistance of LAB isolated from fermented sausages has been reported in many studies (Fraqueza, 2015, for a review). Recently, the antibiotic resistance in *Lactobacillus* spp. was assessed by comparing phenotypes and genotypes based on antibioresistance genes (Campedelli et al., 2019). Some antibiotic resistance genes identified in LAB are transferable and can be potentially transferred between LAB *in vitro* or in model rumen and plant environments and fermented milk (Toomey et al., 2009a,b).

However, knowledge about the capacity of LAB to transfer antibiotic resistance in meat matrices is quite limited. During sausage fermentation, the transfer of plasmids carrying antibiotic resistance was demonstrated between strains of *L. curvatus* (Vogel et al., 1992). A very high frequency of plasmid transfer was also observed between strains of *Enterococcus faecalis* during the ripening of fermented sausages showing that sausages facilitate close contact between strains and so genetic transfer (Cocconcelli et al., 2003). The spread of antibiotic resistance also concerns meat-associated CNS (Mauriello et al., 2000; Martín et al., 2006; Resch et al., 2008; Even et al., 2010; Marty et al., 2012; Chajęcka-Wierzchowska et al., 2015; Osman et al., 2016). Despite the importance of this group of ubiquitous bacteria, to our knowledge, no study has been made of the transfer of antibiotic resistance in food conditions. Among CNS, *S. xylosus* has a long history of safe status as found in most naturally fermented meat products and used as starter culture. However, potentially transferable antibiotic resistances, notably erythromycin, penicillin, and tetracycline, have been reported in *S. xylosus* from fermented meat products (Mauriello et al., 2000; Kastner et al., 2006; Martín et al., 2006; Resch et al., 2008; Even et al., 2010; Marty et al., 2012; Chajęcka-Wierzchowska et al., 2015).

In the present study, the transfer of the tetracycline resistance gene among *S. xylosus* was studied *in vitro* or in fermented sausages, since laboratory transfer experiments do not mimic *in situ* conditions. An estimated conjugation frequency of approximately 1.4 × 10^**−**7^ per recipient under *in situ* conditions was calculated, which was higher than that observed in our filter mating conditions. In the only study concerning the transfer of tetracycline resistance in fermented sausages between *E. faecalis* strains, a very high frequency of 10^−3^ per recipient was observed, superior to filter mating (Cocconcelli et al., 2003). In fact, variable rates of plasmid transfer, from 10^−2^ to 10^−9^, are given in the literature. Factors influencing this variation were identified in a meta-analysis based on 28 articles (Hunter et al., 2008). This analysis revealed highly significant associations between transfer frequency and donor and recipient genera. The nature of the transferable genetic element and the membership of the same genus of donor and recipient strains also significantly influenced the transfer (Hunter et al., 2008).

The gene encoding tetracycline resistance is frequently found on small plasmids common among staphylococci (Schwarz and Noble, 1994; Perreten et al., 1998). Small transmissible plasmids carrying the gene *tetK* encoding efflux protein represent a family of closely related plasmids ranging from 4.4 to 4.7 kb (Chopra and Roberts, 2001). In our study, the *tetK* gene of the *S. xylosus* strain XIV 10B1 isolated from sausage was transferable to a *S. xylosus* recipient and was located on a pT181-like plasmid, the *S. aureus* plasmid pT181 being the prototype from the staphylococcal tetracycline resistance plasmid family (Khan and Novick, 1983). These pT181-like plasmids belonged to the pMV158 superfamily of small mobilizable plasmids (Francia et al., 2004). These non-conjugative mobilizable plasmids are not able to transfer on their own but are able to exploit the mating pore encoded by a helper element, conjugative plasmids or transposons (Francia et al., 2004; Ramsay et al., 2016). In staphylococci, three mechanisms of conjugative mobilization have been elucidated by (1) encoding a mimic sequence of the conjugative plasmid *oriT*, (2) encoding a distinct relaxase (Mob) compatible with the mating pore and its own related *oriT*, or (3) carrying a compatible replicative relaxase (Rep) (Ramsay et al., 2016). The helper element by which the *S. xylosus* XV 10B1 mobilizable plasmid is transferred between *S. xylosus* strains remains to be identified.

Metagenomic analyses have been applied to monitor antibiotic resistance genes in two dairy products microbiota. One screened antibiotic resistance genes in mozzarella cheese (Devirgiliis et al., 2014) and the other in raw milk and blue veined cheese (Flórez et al., 2017). The latter study targeted on tetracycline resistance genes coding for ribosomal protection proteins (*tetM, tetS*) and efflux pumps (*tetA, tetL*); *tetA* was found in plasmids from Gram-negative bacteria, while the three others were from lactic acid bacteria (Flórez et al., 2017). However, the dairy microbiota “resistome” and even more the meat microbiota “resistome” have yet to be explored. The antimicrobial resistance of these food bacteria could be transmitted to gut microbiota and constitute a threat to human health (Devirgiliis et al., 2011; Schjørring and Krogfelt, 2011; Forslund et al., 2014).

The transfer of antimicrobial resistance between starters and foodborne pathogens has to be further investigated, to our knowledge, transfer occurred *in vitro* between LAB and *Listeria* but not with other pathogens and not in cheese matrix (Gevers et al., 2003; Toomey et al., 2009b). Similar studies should be carried out to assess if fermented sausages can constitute an environment for gene exchange between starters and pathogenic bacteria, it would be of utmost importance between *S. xylosus* and *S. aureus* or other CNS as plasmids of the pT181 family are transmissible and could disseminated within fermented foods. Thus, the selection of starter cultures such as *S. xylosus* should consider antibiotic resistances and their potential transfer to other strains. All these studies have pushed the starter industry to consider this risk factor and therefore comply with the AFSSA safety recommendations and the European QPS approach.

## Author Contributions

SL and RT conceived the study and wrote the manuscript. SL, SC, and RT designed the experiments. SL and SC performed the laboratory experiments. SL, SC, and RT analyzed the data, contributed to preparing the final version of the manuscript, and approved the final manuscript.

### Conflict of Interest Statement

The authors declare that the research was conducted in the absence of any commercial or financial relationships that could be construed as a potential conflict of interest.

## References

[ref1] Anisimova E. Yarullina D. (2018). Characterization of erythromycin and tetracycline resistance in *Lactobacillus fermentum* strains. Int. J. Microbiol. 2018:3912326. 10.1155/2018/3912326 30534155 PMC6252201

[ref2] Bhargava K. Zhang Y. (2012). Multidrug-resistant coagulase-negative Staphylococci in food animals. J. Appl. Microbiol. 113, 1027–1036. 10.1111/j.1365-2672.2012.05410.x 22816491

[ref3] Campedelli I. Mathur H. Saletti E. Clarke S. Rea M. C. Torriani S. (2019). Genus-wide assessment of antibiotic resistance in *Lactobacillus* spp. Appl. Environ. Microbiol. 85:e01738-18. 10.1128/AEM.01738-18 PMC6293106 30366997

[ref4] CA-SFM/EUCAST. (2017). Comité de l’Antibiogramme de la Société Française de Microbiologie. (Paris, France: Société Française de Microbiologie Ed), 1–127. http://www.sfm-microbiologie.org/UserFiles/files/casfm/

[ref5] Chajęcka-Wierzchowska W. Zadernowska A. Nalepa B. Sierpińska M. Łaniewska-Trokenheim Ł. (2015). Coagulase-negative staphylococci (CoNS) isolated from ready-to-eat food of animal origin-phenotypic and genotypic antibiotic resistance. Food Microbiol. 46, 222–226. 10.1016/j.fm.2014.08.001 25475289

[ref6] Chon J. W. Jung H. I. Kuk M. Lim J. S. Seo K. H. Kim S. K. (2016). Microbiological evaluation of pork and chicken by-products in South Korea. J. Food Prot. 79, 715–722. 10.4315/0362-028X.JFP-15-395 27296417

[ref7] Chopra I. Roberts M. (2001). Tetracycline antibiotics: mode of action, applications, molecular biology, and epidemiology of bacterial resistance. Microbiol. Mol. Biol. Rev. 65, 232–260. 10.1128/MMBR.65.2.232-260.2001 11381101 PMC99026

[ref8] Cocconcelli P. S. Cattivelli D. Gazzola S. (2003). Gene transfer of vancomycin and tetracycline resistance among *E. faecalis* during cheese and sausage fermentations. Int. J. Food Microbiol. 88, 315–323. 10.1016/S0168-1605(03)00194-6 14597004

[ref9] Corbière Morot-Bizot S. Talon R. Leroy S. (2004). Development of a multiplex PCR for the identification of *Staphylococcus* genus and four staphylococcal species isolated from food. J. Appl. Microbiol. 97, 1087–1094. 10.1111/j.1365-2672.2004.02399.x 15479426

[ref10] Coton E. Desmonts M. H. Leroy S. Coton M. Jamet E. Christieans S. (2010). Biodiversity of coagulase-negative staphylococci in French cheeses, dry fermented sausages, processing environments and clinical samples. Int. J. Food Microbiol. 137, 221–229. 10.1016/j.ijfoodmicro.2009.11.023 20061042

[ref11] Devirgiliis C. Barile S. Perozzi G. (2011). Antibiotic resistance determinants in the interplay between food and gut microbiota. Genes Nutr. 6, 275–284. 10.1007/s12263-011-0226-x 21526400 PMC3145056

[ref12] Devirgiliis C. Zinno P. Stirpe M. Barile S. Perozzi G. (2014). Functional screening of antibiotic resistance genes from a representative metagenomic library of food fermenting microbiota. Biomed. Res. Int. 2014:290967. 10.1155/2014/290967 25243126 PMC4163480

[ref13] Ed-Dra A. Filali F. R. Bouymajane A. Benhallam F. El Allaoui A. Chaiba A. (2018). Antibiotic susceptibility of *S. aureus* isolated from sausages in Meknes, Morocco. Vet. World 11, 1459–1465. 10.14202/vetworld.2018.1459-1465 30532502 PMC6247881

[ref14] Even S. Leroy S. Charlier C. Ben Zakour N. Chacornac J. P. Lebert I. (2010). Low occurrence of safety hazards in coagulase negative staphylococci isolated from fermented foodstuffs. Int. J. Food Microbiol. 139, 87–95. 10.1016/j.ijfoodmicro.2010.02.019 20226555

[ref15] Flórez A. B. Vázquez L. Mayo B. (2017). A Functional metagenomic analysis of tetracycline resistance in cheese bacteria. Front. Microbiol. 8:907. 10.3389/fmicb.2017.00907 28596758 PMC5442184

[ref16] Forslund K. Sunagawa S. Coelho L. P. Bork P. (2014). Metagenomic insights into the human gut resistome and the forces that shape it. BioEssays 36, 316–329. 10.1002/bies.201300143 24474281

[ref17] Francia M. V. Varsaki A. Garcillán-Barcia M. P. Latorre A. Drainas C. de la Cruz F. (2004). A classification scheme for mobilization regions of bacterial plasmids. FEMS Microbiol. Rev. 28, 79–100. 10.1016/j.femsre.2003.09.001 14975531

[ref18] Fraqueza M. J. (2015). Antibiotic resistance of lactic acid bacteria isolated from dry-fermented sausages. Int. J. Food Microbiol. 212, 76–88. 10.1016/j.ijfoodmicro.2015.04.035 26002560

[ref19] Gevers D. Huys G. Swings J. (2003). In vitro conjugal transfer of tetracycline resistance from *Lactobacillus* isolates to other Gram-positive bacteria. FEMS Microbiol. Lett. 225, 125–130. 10.1016/S0378-1097(03)00505-6 12900030

[ref20] Götz F. Zabielski J. Philipson L. Lindberg M. (1983). DNA homology between the arsenate resistance plasmid pSX267 from *S. xylosus* and the penicillinase plasmid pI258 from *S. aureus*. Plasmid 9, 126–137. 10.1016/0147-619X(83)90015-X 6602348

[ref21] Greppi A. Ferrocino I. La Storia A. Rantsiou K. Ercolini D. Cocolin L. (2015). Monitoring of the microbiota of fermented sausages by culture independent rRNA-based approaches. Int. J. Food Microbiol. 212, 67–75. 10.1016/j.ijfoodmicro.2015.01.016 25724303

[ref22] Huang X. Madan A. (1999). CAP3: A DNA sequence assembly program. Genome Res. 9, 868–877. 10.1101/gr.9.9.868 10508846 PMC310812

[ref23] Hunter P. R. Wilkinson D. C. Catling L. A. Barker G. C. (2008). Meta-analysis of experimental data concerning antimicrobial resistance gene transfer rates during conjugation. Appl. Environ. Microbiol. 74, 6085–6090. 10.1128/AEM.01036-08 18708517 PMC2565951

[ref24] Kastner S. Perreten V. Bleulera H. Hugenschmidt G. Lacroix C. Meile L. (2006). Antibiotic susceptibility patterns and resistance genes of starter cultures and probiotic bacteria used in food. Syst. Appl. Microbiol. 29, 145–155. 10.1016/j.syapm.2005.07.009 16464696

[ref25] Khan S. A. Novick R. P. (1983). Complete nucleotide sequence of pT181, a tetracycline-resistance plasmid from *S. aureus*. Plasmid 10, 251–259. 10.1016/0147-619X(83)90039-2 6657777

[ref26] Leroy S. Giammarinaro P. Chacornac J. P. Lebert I. Talon R. (2010). Biodiversity of indigenous staphylococci of naturally fermented dry sausages and manufacturing environments of small-scale processing units. Food Microbiol. 27, 249–301. 10.1016/j.fm.2009.11.005 20141949

[ref27] Li L. Ye L. Zhang S. Meng H. (2016). Isolation and identification of aerobic bacteria carrying tetracycline and sulfonamide resistance genes obtained from meat processing plant. J. Food Sci. 81, M1480–M1484. 10.1111/1750-3841.13318 27100915

[ref28] Lüdin P. Roetschi A. Wüthrich D. Bruggmann R. Berthoud H. Shani N. (2018). Update on tetracycline susceptibility of *Pediococcus acidilactici* based on strains isolated from swiss cheese and whey. J. Food Prot. 81, 1582–1589. 10.4315/0362-028X.JFP-18-160 30169118

[ref29] Malik S. Peng H. Barton M. D. (2005). Antibiotic resistance in staphylococci associated with cats and dogs. J. Appl. Microbiol. 99, 1283–1293. 10.1111/j.1365-2672.2005.02699.x 16313400

[ref30] Marmur J. (1961). A procedure for the isolation of deoxyribonucleic acid from microorganism. J. Mol. Biol. 3, 208–218. 10.1016/S0022-2836(61)80047-8

[ref31] Martín B. Garriga M. Hugas M. Bover-Cid S. Veciana-Nogues M. T. Aymerich T. (2006). Molecular, technological and safety characterization of Gram-positive catalase-positive cocci from slightly fermented sausages. Int. J. Food Microbiol. 107, 148–158. 10.1016/j.ijfoodmicro.2005.08.024 16297478

[ref32] Marty E. Bodenmann C. Buchs J. Hadorn R. Eugster-Meier E. Lacroix C. (2012). Prevalence of antibiotic resistance in coagulase-negative staphylococci from spontaneously fermented meat products and safety assessment for new starters. Int. J. Food Microbiol. 159, 74–83. 10.1016/j.ijfoodmicro.2012.07.025 23072691

[ref33] Mathur S. Singh R. (2005). Antibiotic resistance in food lactic acid bacteria -a review. Int. J. Food Microbiol. 105, 281–295. 10.1016/j.ijfoodmicro.2005.03.008 16289406

[ref34] Mauriello G. Moschetti G. Villani F. Blaiotta G. Coppola S. (2000). Antibiotic resistance of coagulase-negative staphylococci isolated from artisanal Naples-type salami. Int. J. Food Sci. Nutr. 51, 19–24. 10746101 10.1080/096374800100868

[ref35] Morot-Bizot S. Talon R. Leroy-Setrin S. (2003). Development of specific PCR primers for a rapid and accurate identification of *S. xylosus*, a species used in food fermentation. J. Microbiol. Methods 55, 279–286. 10.1016/S0167-7012(03)00159-3 14500019

[ref36] Osman K. Badr J. Al-Maary K. S. Moussa I. M. I. Hessain A. M. Girah Z. M. S. A. Abo-shama U. H. Orabi A. Saad A. (2016). Prevalence of the antibiotic resistance genes in coagulase-positive-and negative-*Staphylococcus* in chicken meat retailed to consumers. Front. Microbiol. 7:1846. 10.3389/fmicb.2016.01846 27920760 PMC5118462

[ref37] Perreten V. Giampà N. Schuler-Schmid U. Teuber M. (1998). Antibiotic resistance genes in coagulase-negative staphylococci isolated from food. Syst. Appl. Microbiol. 21, 113–120. 10.1016/S0723-2020(98)80014-3 9741115

[ref38] Ramsay J. P. Kwong S. M. Murphy R. J. T. Yui Eto K. Price K. J. Nguyen Q. T. (2016). An updated view of plasmid conjugation and mobilization in *Staphylococcus*. Mob. Genet. Elem. 6:e1208317. 10.1080/2159256X.2016.1208317 PMC4993578 27583185

[ref39] Ratsimba A. Leroy S. Chacornac J. P. Rakoto D. Arnaud E. Jeannoda V. (2017). Staphylococcal ecosystem of kitoza, a traditional Malagasy meat product. Int. J. Food Microbiol. 246, 20–24. 10.1016/j.ijfoodmicro.2017.02.001 28187327

[ref40] Resch M. Nagel V. Hertel C. (2008). Antibiotic resistance of coagulase-negative staphylococci associated with food and used in starter cultures. Int. J. Food Microbiol. 127, 99–104. 10.1016/j.ijfoodmicro.2008.06.013 18625535

[ref41] Roberts M. C. (1996). Tetracycline resistant determinants: mechanisms of action, regulation of expression, genetic mobility and distribution. FEMS Microbiol. Rev. 19, 1–24. 10.1111/j.1574-6976.1996.tb00251.x 8916553

[ref42] Sánchez Mainar M. Stavropoulou D. A. Leroy F. (2017). Exploring the metabolic heterogeneity of coagulase-negative staphylococci to improve the quality and safety of fermented meats: a review. Int. J. Food Microbiol. 247, 24–37. 10.1016/j.ijfoodmicro.2016.05.021 27234590

[ref43] Schjørring S. Krogfelt K. A. (2011). Assessment of bacterial antibiotic resistance transfer in the gut. Int. J. Microbiol. 2011:312956. 10.1155/2011/312956 21318188 PMC3034945

[ref44] Schwarz S. Noble W. C. (1994). Tetracycline resistance in staphylococci from the skin of pigs. J. Appl. Bacteriol. 78, 320–326. 10.1111/j.1365-2672.1994.tb01635.x 8200858

[ref45] Schwarz S. Roberts M. C. Werckenthin C. Pang Y. Lange C. (1998). Tetracycline resistance in *Staphylococcus* spp. from domestic and pet animals. Vet. Microbiol. 63, 217–228. 10.1016/S0378-1135(98)00234-X 9851000

[ref46] Simeoni D. Rizzottia L. Cocconcelli P. Gazzola S. Dellaglioa F. Torriani S. (2008). Antibiotic resistance genes and identification of staphylococci collected from the production chain of swine meat commodities. Food Microbiol. 25, 196–201. 10.1016/j.fm.2007.09.004 17993395

[ref47] Strommenger B. Kettlitz C. Werner G. Witte W. (2003). Multiplex PCR assay for simultaneous detection of nine clinically relevant antibiotic resistance genes in *S. aureus*. J. Clin. Microbiol. 41, 4089–4094. 10.1128/JCM.41.9.4089-4094.2003 12958230 PMC193808

[ref48] Talon R. Leroy S. (2011). Diversity and safety hasards of bacteria involved in meat fermentations. Meat Sci. 89, 303–309. 10.1016/j.meatsci.2011.04.029 21620574

[ref49] Thaker M. Spanogiannopoulos P. Wright G. D. (2010). The tetracycline resistome. Cell. Mol. Life Sci. 67, 419–431. 10.1007/s00018-009-0172-6 19862477 PMC11115633

[ref50] Toomey N. Monaghan A. Fanning S. Bolton D. (2009a). Transfer of antibiotic resistance marker genes between lactic acid bacteria in model rumen and plant environments. Appl. Environ. Microbiol. 75, 3146–3152. 10.1128/AEM.02471-08 19270126 PMC2681641

[ref51] Toomey N. Monaghan A. Fanning S. Bolton D. (2009b). Assessment of antimicrobial resistance transfer between lactic acid bacteria and potential foodborne pathogens using in vitro methods and mating in food matrix. Foodborne Pathog. Dis. 6, 925–933. 10.1089/fpd.2009.0278 19799525

[ref52] van Hoek A. H. A. M. Mevius D. Guerra B. Mullany P. Roberts A. P. Aarts H. J. M. (2011). Acquired antibiotic resistance genes: an overview. Front. Microbiol. 2:203. 10.3389/fmicb.2011.00203 22046172 PMC3202223

[ref53] Verraes C. van Boxstael S. van Meervenne E. van Coillie E. Butaye P. Catry B. (2013). Antimicrobial resistance in the food chain: a review. Int. J. Environ. Res. Public Health 10, 2643–2669. 10.3390/ijerph10072643 23812024 PMC3734448

[ref54] Vogel R. F. Becke-Schmid M. Entgens P. Hammes W. P. (1992). Plasmid transfer and segregation in *L. curvatus* LTH 1432 *in vitro* and during sausage fermentation. Syst. Appl. Microbiol. 15, 129–136. 10.1016/S0723-2020(11)80149-9

[ref55] Witte W. (1998). Medical consequences of antibiotic use in agriculture. Science 279, 996–997. 10.1126/science.279.5353.996 9490487

[ref56] Wu S. Huang J. Wu Q. Zhang J. Zhang F. Yang X. (2018). *S. aureus* isolated from retail meat and meat products in china: incidence, antibiotic resistance and genetic diversity. Front. Microbiol. 9:2767. 10.3389/fmicb.2018.02767 30498486 PMC6249422

